# Functional evaluation of pure natural edible Ferment: protective function on ulcerative colitis

**DOI:** 10.3389/fmicb.2024.1367630

**Published:** 2024-06-17

**Authors:** Yanjun Chen, Shengzhi Ye, Jiaolong Shi, Hao Wang, Guangxu Deng, Guangxian Wang, Shijie Wang, Qingbin Yuan, Lunan Yang, Tingyu Mou

**Affiliations:** ^1^Department of General Surgery & Guangdong Provincial Key Laboratory of Precision Medicine for Gastrointestinal Cancer, Nanfang Hospital, Southern Medical University, Guangzhou, China; ^2^First Department of Gastrointestinal Surgery, Hainan General Hospital, Hainan Affiliated Hospital of Hainan Medical University, Haikou, China; ^3^Department of Gastrointestinal and Anorectal, The First People’s Hospital of Zhaoqing, Zhaoqing, China; ^4^Jiangsu Biodep Biotechnology Co., Ltd., Jiangyin, China; ^5^College of Foods Science and Biology, Hebei University of Science and Technology, Shijiazhuang, China; ^6^Junlebao Dairy Group Co., Ltd., Shijiazhuang, China; ^7^Department of Plastic and Aesthetic Surgery, Nanfang Hospital, Southern Medical University, Guangzhou, China

**Keywords:** ulcerative colitis (UC), lactic acid bacteria, Ferment, 16S rRNA, animal experiment

## Abstract

**Purpose:**

To investigate the therapeutic efficiency of a novel drink termed “Ferment” in cases of ulcerative colitis (UC) and its influence on the gut microbiota.

**Method:**

In this study, we developed a complex of mixed fruit juice and lactic acid bacteria referred to as Ferment. Ferment was fed to mice for 35 days, before inducing UC with Dextran Sulfate Sodium Salt. We subsequently investigated the gut microbiome composition using 16S rRNA sequencing.

**Result:**

After Ferment treatment, mouse body weight increased, and animals displayed less diarrhea, reduced frequency of bloody stools, and reduced inflammation in the colon. Beneficial bacteria belonging to *Ileibacterium*, *Akkermansia*, and *Prevotellacea* were enriched in the gut after Ferment treatment, while detrimental organisms including *Erysipelatoclostridium*, *Dubosiella*, and *Alistipes* were reduced.

**Conclusion:**

These data place Ferment as a promising dietary candidate for enhancing immunity and protecting against UC.

## 1 Introduction

Ulcerative colitis (UC) is a chronic disease characterized by extensive inflammation of the colon (Kaser et al., 2010). UC alternates between states of exacerbation and remission, and one major therapeutic aim is to maintain periods of remission (Ungaro et al., 2019). The current pharmacological treatment strategies include application of aminosalicylates, corticosteroids, immunosuppressants, antibiotics, and biological agents (Bernstein, 2015). In recent years, anti-TNF therapy has emerged as a major treatment for moderate to severe UC and Crohn’s disease (CD) (Chudy-Onwugaje et al., 2019). However, these aforementioned treatments have disadvantages such as long-term application of corticosteroids leading to hypokalemia, hypertension, abnormal blood sugar metabolism, and water/sodium retention (Magro et al., 2020). Furthermore, immunosuppressants such as methotrexate may cause gastrointestinal reactions, liver damage, bone marrow suppression, and allergies (Damião et al., 2019). As such, an effective treatment for UC with limited off-target effects remains elusive.

Over the past 20 years, researchers have focused on the role of probiotics in modulating inflammatory bowel disease (IBD). Probiotics were shown to restore mucosal barrier function (Dicksved et al., 2012), and correct imbalances in the intestinal microbiota (Thomas et al., 2005). Probiotics effectively induce and maintain remission in UC patients, suggesting that optimizing composition of the gut microbiota is a useful strategy for UC management. Lactic acid bacteria (LAB) are major probiotics, non-toxic, and beneficial to the human body. Live *Lactobacilli* improve symptoms of enteritis by inhibiting inflammation in intestinal epithelial cells. Some LAB strains can stimulate the immune system (monocytes and macrophages) to drive a homeostatic balance between production of pro-inflammatory and anti-inflammatory cytokines such as IL-10, thereby regulating intestinal inflammation (Ma et al., 2021). Many LAB have been used in food or drug applications, including *Lactobacillus plantarum*, *Lactobacillus rhamnosus* and *Bifidobacterium lactis* (Oelschlaeger, 2010), but these probiotics display insufficient efficacy when applied alone. The limitations of single bacterial strains as a probiotic include patient intolerance, a narrow spectrum of biological activities, and individual variations that promote unstable therapeutic effects (Hemarajata and Versalovic, 2013; Kechagia et al., 2013; Saez-Lara et al., 2015). These observations indicate that further study is required to utilize LAB as an effective therapy.

Recent studies of fruit and vegetable extracts have highlighted their utility in the management of inflammation-related diseases. Chinese white pears contain high concentrations of flavonoids (Liu et al., 2021), which have multiple beneficial effects on human health, including inhibition of malignant bacterial proliferation, inflammation, cancer growth, oxidation, and thrombosis (Yu-Min et al., 2020). Furthermore, prostaglandin E2 production in the murine Dextran Sulfate Sodium Salt (DSS)-induced colitis model can be reduced by ingestion of Aronia Berry extract, which also reduced levels of nitric oxide, IL-6, and TNF-α in macrophages, leading to improved clinical symptoms of enteritis (Kang et al., 2017). Red heart pitaya extract also significantly reduced levels of IL-6, IL-1β, TNF-α, and increased levels of IL-10 in the serum of mice. Lastly, green cabbage is rich in glucosinolates (Alexandre et al., 2020), the degradation products of which have antibacterial, anti-inflammatory, and anti-cancer activities (Wu et al., 2021). After ingesting a compound fruit and vegetable fermented juice, both the composition of the intestinal flora, and damage to the intestinal mucosa improved in patients with irritable bowel syndrome (IBS) (Singh et al., 2014; Yang et al., 2016).

In this study, we mixed and fermented three LAB with compound fruit and vegetable juice to produce a beverage, hereafter referred to as “Ferment.” Ferment demonstrated probiotic effects, which help restore the balance of gut bacteria in individuals with UC. Secondly, Ferment contains high levels of antioxidants and other anti-inflammatory compounds, which may reduce inflammation in the gut. Thirdly, Ferment is nutritious, containing vitamins, minerals, and other nutrients, which may help support overall health and well-being. As fruits and LAB are naturally occurring, we predict Ferment is less likely to promote the side effects observed with other conventional medications used to treat UC. In conclusion, Ferment is a safe, inexpensive, and convenient treatment option for UC.

## 2 Materials and methods

### 2.1 Animals and reagents

Four-week-old male C57BL/6 mice were purchased from the Experimental Animal Center of Guangdong Province (Guangdong, China), and were bred at the Experimental Animal Center of Nanfang Hospital. Animals were fed standard chow pellets disinfected by ultraviolet light, had access to purified water supplied in bottles, and were acclimatized for 14 days prior to experimentation. During these 2 weeks, mice were randomly mixed weekly to ensure comparable microbiota compositions between animals. DSS was purchased from MP Biomedicals (Santa Ana, CA, United States). All experiments were approved by the Ethics Committee of Experimental Animals, Nanfang Hospital, Southern Medical University and were performed in accordance with the guidelines for the ethical treatment of animals.

### 2.2 Preparation of Ferment

#### 2.2.1 Operating procedures

**Figure 1:**
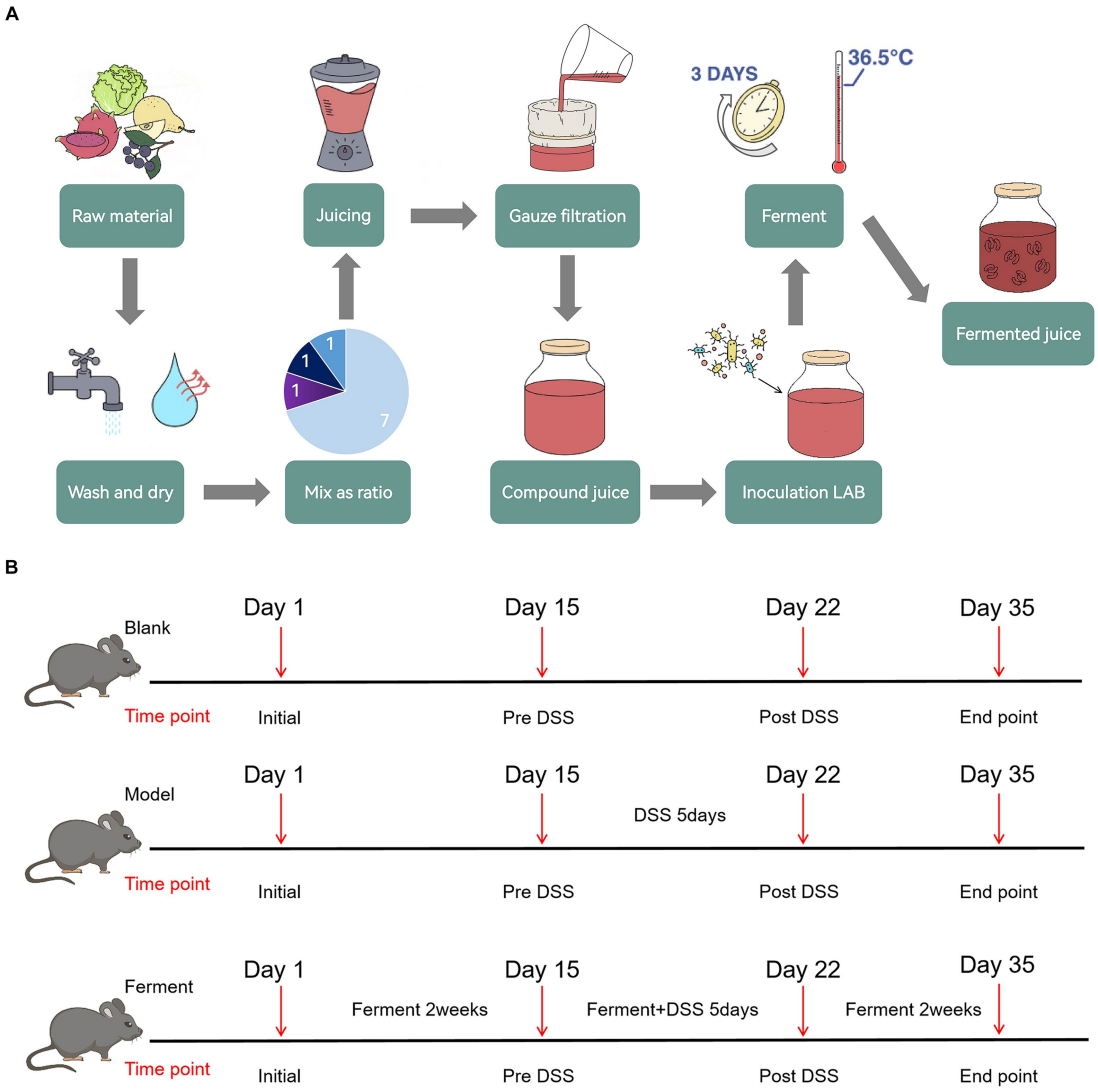
Schematic illustration of Ferment use in ulcerative colitis treatment **(A)** Ferment production flow chart. **(B)** Experimental protocol flow chart.

Raw fruits and vegetables were first washed and dried separately, before being weighed and mixed in defined ratios and juiced. The juice was subsequently filtered through three layers of gauze to obtain a compound juice. Mixed LAB were used to inoculated the compound juice before fermenting, yielding the final product, Ferment (Figure 1A).

#### 2.2.2 Ratio of fruits and vegetables

Chinese white pears were the major raw material, and were mixed with Aronia berries, red heart pitayas, and green cabbage. The number of viable bacteria and subsequent acid production in the fermented juice were quantified and analyzed as assessment indicators. Orthogonal experiments were conducted three times to obtain an optimized ratio of the raw materials. The final composition of Ferment was 70 g/100 mL of Chinese white pear, 10 g/100 mL Aronia berries, 10 g/100 mL of red pitaya, and 10 g/100 mL of green cabbage. Using this ratio, the number of viable bacteria in Ferment was 4.81 × 10^8^ CFU/mL, and associated acid production was 6.53 g/kg.

#### 2.2.3 Ratio of compound LAB

*Bifidobacterium lactis* Bla019, *Lactobacillus rhamnosus* LR05, and *Lactobacillus plantarum* were used to inoculate the juice. The number of viable bacteria and associated acid production were used as indicators. Sugar (6%) was added to the compound juice, along with 4% of the compound LAB inoculum, and the juice was subsequently cultured at 36°C for 3 days. To determine an optimized ratio of LAB, we sequentially altered the viable concentration of each individual organism. The final concentration of each organism was 10^7^ CFU/mL in an equal ratio. The total number of viable bacteria in the fermented juice was 4.36 × 10^8^ CFU/mL, and the acid production was 6.35 g/kg.

#### 2.2.4 Optimization of Ferment

Acid content and Superoxide Dismutase (SOD) activity in the fermented juice was determined to optimize the composition of Ferment. We found that the optimal composition of Ferment consisted of 4.6% bacterial inoculum, and 6% sugar, with a fermentation temperature of 36.5°C and a fermentation time of 3 days. Acid production in Ferment was 7.01 g/kg, and SOD enzyme activity was 429.57 U/mL.

#### 2.2.5 Chemical composition of Ferment

We used a chemical reagent method to determine the composition Ferment. The content of flavonoids was 4.72 mg/mL, polyphenols were 0.25 mg/mL, tannins were 296.72 mg/L, anthocyanins were 52.18 mg/L and glucosinolates were 2.38 mg/g (Supplementary Table S1). The total amount of 10 organic acids was characterized by high-performance liquid chromatography (HPLC) as 3.03 g/L, and the most abundant compound was oxalic acid (Supplementary Table S2). Sixteen volatile substances were also quantified by HP-SPME-GC-MS. Esters were the major volatile substance, accounting for 65.95% of the total (Supplementary Table S3). Finally, the 10 polyphenols detected by UHPLC-MS were dominated by astilbin with a concentration of 1.55 μg/mL (Supplementary Table S4).

### 2.3 Experimental UC and drug administration

To evaluate the efficacy of Ferment in treatment of UC, after 2 weeks of acclimatization and cage mixing, 19 C57BL/6 specific pathogen free mice were randomly divided into three groups: seven mice in the control group, six mice in the DSS colitis group, and six mice in Ferment group. Mice were fed with a normal diet throughout the 35-day experiment. The Ferment group were additionally administered 10 μL/g body weight of Ferment intragastrically once daily. Mice were subsequently administered 2.5% DSS solution on the 15th day for 5 days. Two days after the DSS treatment course (the 22nd day), three mice in each group were randomly selected to undergo endoscopy, and were sacrificed for intestine and colon tissue collection. On the 35th day, all remaining mice received endoscopy and were sacrificed for tissue collection (Figure 1B). Mice were weighed daily, and their stools were frequently sampled. After administration of DSS, the pathological features of each mouse including rectal bleeding, rectal prolapse, stool consistency, and the presence of bloody stool were recorded in accordance with the inflammation score (Kim et al., 2012).

### 2.4 Histopathology

Intestinal and colonic tissues were fixed in 10% neutral formalin buffer for 24 h before transferring to 70% ethanol, and embedding in paraffin. Samples were cut into 5 μm thick sections and stained with hematoxylin and eosin, before pathology was evaluated blind, and scored. The severity of intestinal mucosal injury was determined from stained sections. Inflammation was scored based on epithelial damage, inflammatory cell infiltration, crypt loss, and goblet cell reduction (Jang et al., 2019).

### 2.5 Microbiome analysis

All fecal samples were stored immediately at −80°C following collection. Samples were analyzed at day 1, day 14 (immediately prior to DSS administration) and day 22 (post DSS). At the end of the experiment, fecal samples were subject to high-throughput 16S rDNA sequencing (Guangdong Magigene Biotechnology Co, Ltd. Guangzhou, China). Amplicon libraries covering the V3–V4 hypervariable regions of the universal bacterial 16S rRNA gene were prepared by PCR. The PCR reaction volume was 50 μL, and the products were imaged, purified, and quantified after amplification. The NEBNext^®^ Ultra^™^ II DNA Library Prep Kit for Illumina^®^ (New England Biolabs, United States) was used to construct a PE library. The Illumina NovaSeq 6000 PE250 platform was used for sequencing. The raw reads were quality controlled using Fastp v0.14.1 (-W 4 -M 20). The clean reads were merged using usearch-fastq_mergepairs (v10), and merged sequences were subject to further quality control using Fastp v0.14.1 (-W 4 -M 20). Silva v138 was used for bacterial taxon annotation through amplicon sequence variant (ASV) assignment. ASVs were resolved using the DADA2 denoising method in the QIIME2 software (Bolyen et al., 2019), and the sequences of chimeras were depleted. The MGGICHAND visualization platform^1^ was used to describe correspondence between samples and species. Non-Metric Multidimensional Scaling (NMDS) was conducted using the R package to display the microbiome beta-diversity. A Wilcoxon rank sum test was used to compare the bacterial classification of the two groups at the genus level. The dominance of bacterial communities between groups was analyzed by linear discriminant analysis [LDA, LDA score (Log10) = 3 as the boundary value].

### 2.6 Statistical analyses

All statistical analyses were conducted using R (version 3.4.1.) and GraphPad Prism (version 9.00), and results were validated using SPSS (version 26). Analyze the changes in body weight and inflammation score between mouse groups through one-way ANOVA. And the differences between each two groups were detected using the Wilcoxon test.

## 3 Results

### 3.1 Ferment treatment ameliorates DSS-induced colitis

**Figure 2:**
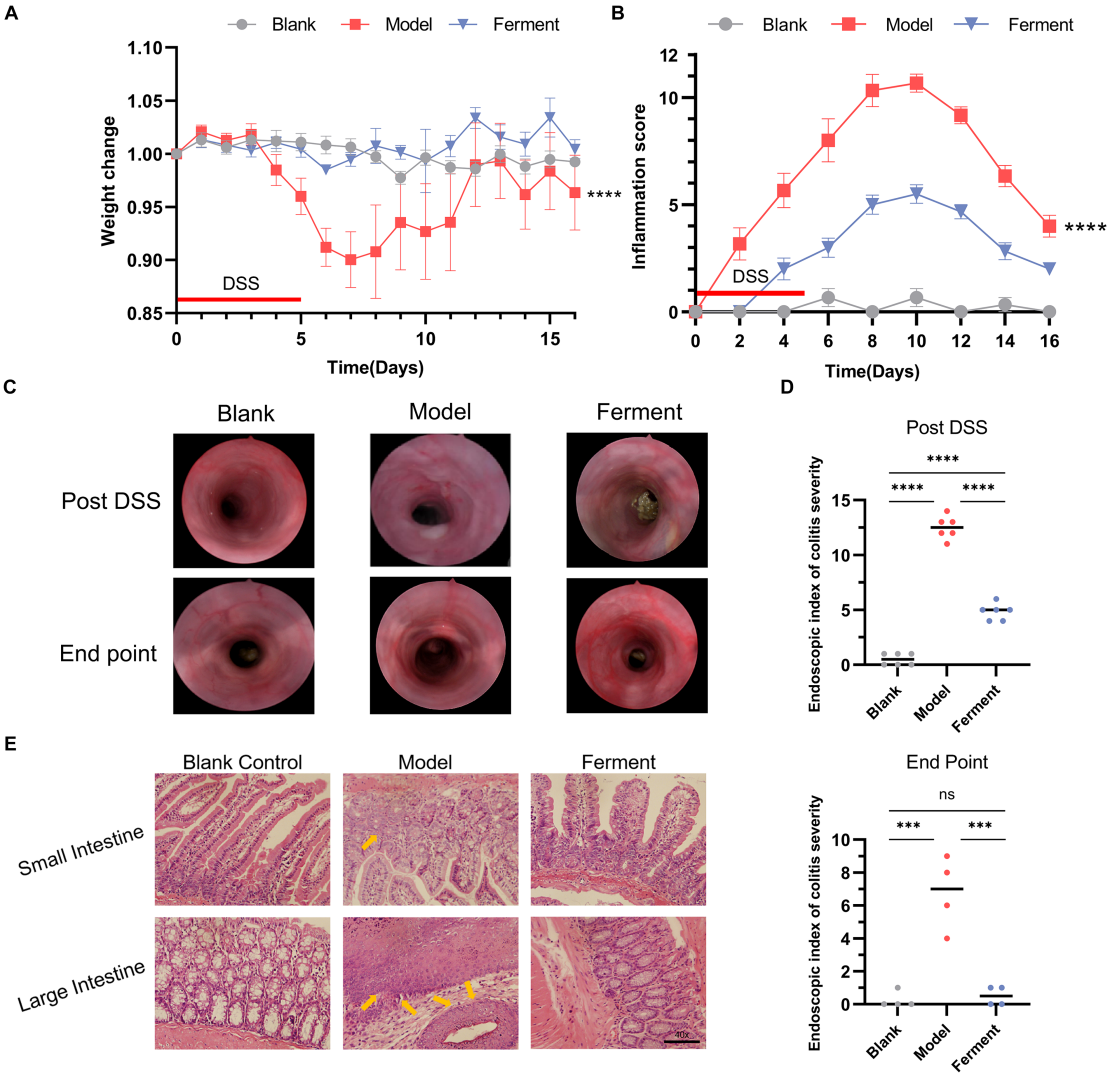
Effect of Ferment and Dextran Sulfate Sodium Salt (DSS) on body weight and intestine structure in mice. **(A)** Percent change of mouse body weight following DSS administration. **(B)** Inflammation score of mice following DSS administration. **(C)** Colonoscopy of three groups of mice post-DSS administration and at the experimental endpoint. **(D)** Colonoscopy score of three groups of mice post-DSS administration and at the experimental endpoint. **(E)** Hematoxylin & eosin staining of colon sections from control, DSS-treated, and Ferment-treated groups. **(F)** Histology score of three groups of mice at the experimental endpoint. ^*^p < 0.05, ^**^p < 0.01, ^***^p < 0.001, and ^****^p < 0.0001.

To evaluate the effect of Ferment treatment on UC, we employed the murine DDS-induced colitis model. There was no statistically significant difference in weight between the Ferment and control groups, whereas the weight of the DSS group was significantly lower (Figure 2A). In the final 5 days of the experiment, the Ferment group gained weight exceeding the control group. Furthermore, the Ferment group presented with less diarrhea and a reduced frequency of bloody stools. Mice in the DSS group displayed dark red or black stool, whereas mice in the Ferment group displayed either red or normal stools. Furthermore, rectal prolapse was less frequent in the Ferment group than in the DSS group, and these observations were reflected by the pathological score (Figure 2B). Enteroscopy revealed limited colonic mucosal bleeding and thickening of the colonic mucosa in the Ferment group (Figure 2C). The mucosa was less granular in the Ferment group than in the DSS group. The DSS group displayed severe fiber exudation, compared with only a mild presentation in the Ferment group. Ferment administration markedly decreased mucosal edema, and reduced the ulcer area and bleeding. Overall, Ferment reduced the endoscopic index of colitis severity compared with DSS alone, consistent with the observed clinical symptoms (Figure 2D).

H&E staining revealed that both the DSS and Ferment groups displayed an inflamed intestine characterized by leukocyte infiltration of the lamina propria, which occasionally extended to the underlying layers. The presence of inflammatory cells, including neutrophils, eosinophils, monocytes, plasma cells, and lymphocytes, served as an indicator of gross inflammation, which consisted of crypt epithelial cell hyperplasia, loss of goblet cells, and the presence of cryptitis, crypt abscesses, and erosions (Figure 2E).

Histological assessment of colon sections revealed presence of a healthy epithelium and normal thickness of the smooth muscle in the Blank group (1.33 ± 0.57), and minimal inflammation in the Ferment group (2.67 ± 1.15). Instead, the DSS group displayed high levels of inflammation (8.33 ± 1.52) compared with the control group. Inflammation in the DSS group was characterized by loss of crypts, changes to epithelial cell integrity, a decrease in the number of goblet cells and a marked infiltration of inflammatory cells. Altogether, Ferment ameliorated the severe inflammation of the colon induced by DSS (Figure 2F).

### 3.2 DSS significantly alters the diversity and composition of the intestinal microflora

**Figure 3:**
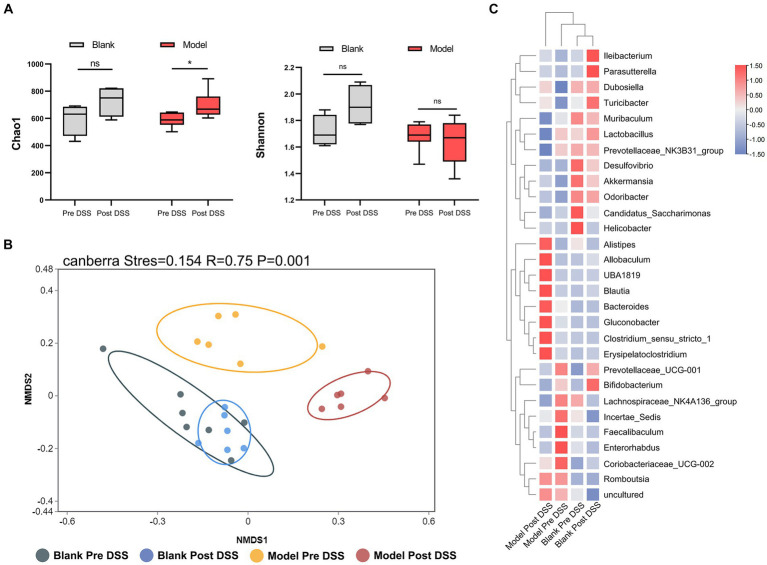
16S rRNA sequencing of fecal bacteria in the Dextran Sulfate Sodium Salt (DSS)-treated group. **(A)** α-diversity Chao1 and Shannon indices in control and DSS-treated animals before and after DSS administration. **(B)** Non-Metric Multidimensional Scaling plot based on Canberra dissimilarity in each sample of control and DSS mice pre and post DSS administration. **(C)** Heatmap showing different bacteria identified in control and DSS mice pre and post DSS administration. ^*^p < 0.05, ^**^p < 0.01, ^***^p < 0.001, and ^****^p < 0.0001.

To investigate changes to the microflora, we performed high-throughput gene sequencing of 16Sr RNA from fecal bacteria of mice in all groups. The pan/core curve revealed a number of common/core species in all samples. The curve reached the platform stage, indicating a suitable sequencing sample size. At the initial time point, we observed no difference in the richness (Chao1) and diversity indices (Shannon) between groups (Supplementary Figure S1A). We next analyzed Non-Metric Multidimensional Scaling (NMDS) between the three groups by Analysis of Similarity (ANOSIM). We determined the NMDS based on the Gower distance between the three groups, which also demonstrated that the composition and structure of the three groups was similar (Supplementary Figure S1B). These analyses demonstrate that there was no statistical difference between the microbiota of the three groups. We next compared the control and DSS groups before and after administering DSS, and identified a distinct difference between the flora of these samples. Considering the α-diversity, during DSS-induced colitis, the richness of the DSS group increased, while the richness of the control group did not change over time (Figure 3A). Furthermore, the NMDS visualization of β-diversity, combined with the results obtained by ANOSIM, indicate that the microbiota structure of the control group did not change over time (*p* > 0.05). The control group was also indistinguishable from the DSS group prior to administration of DSS (*p* > 0.05). However, administration of DSS significantly altered the microbiota composition (*p* = 0.005) (Figure 3B). Post-DSS treatment, *Erysipelatoclostridium*, *Bacteroides* and *Clostridium_sensu_stricto_1* were enriched and became the dominant flora (Figure 3C). Our results indicate that *Bacteroides* were the key bacterial taxa driving intestinal microflora imbalance in the DSS group. Overall, DSS significantly altered the diversity and composition of the intestinal microflora.

### 3.3 Effect of Ferment on normal mice

**Figure 4:**
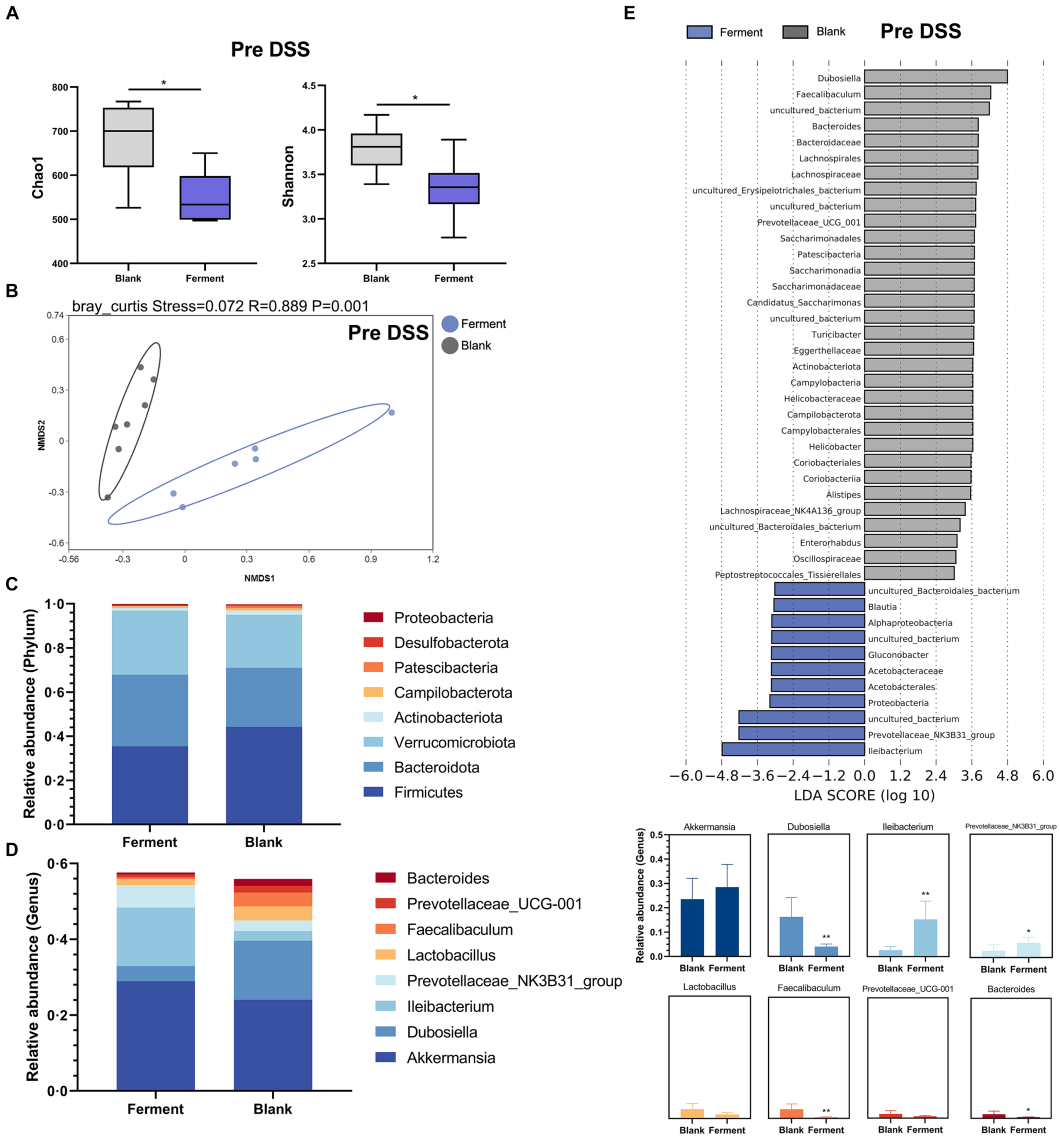
16S rRNA sequencing reveals the protective function of Ferment prior to Dextran Sulfate Sodium Salt (DSS) administration. **(A)** α-diversity Chao1 and Shannon indices in Ferment and control animals prior to DSS administration **(B)** Non-Metric Multidimensional Scaling plot based on Euclidean distance in each sample from control and Ferment mice prior to DSS administration. Effect of Ferment on intestinal flora composition. Gut microbiota composition in Ferment and control animals at the **(C)** phylum and **(D)** genus level. **(E)** Linear discriminant analysis of effect size analysis of characteristic colony search between Ferment and control animals prior to DSS administration. ^*^p < 0.05, ^**^p < 0.01, ^***^p < 0.001, and ^****^p < 0.0001.

Analysis of α-diversity revealed that the richness (Chao1) and Shannon diversity indices of the Ferment group decreased notably prior to administration of DSS (Figure 4A). At the same time point, the β-diversity revealed a clear separation between the control and Ferment groups highlighting a change in microbiota composition (Figure 4B).

Changes to the structure and composition of the microbiota was further evaluated at the phylum and genus level based on sequence data obtained from the Ferment and control groups. We only considered bacteria making up more than 1% relative abundance in each sample. *Firmicutes* was the predominant phylum, making up 45.12 ± 15.99% and 35.46 ± 7.18% of sequences in control and Ferment groups, respectively. *Bacteroidota* made up 26.37 ± 10.89% and 32.93 ± 7.40%, respectively (Figure 4C). The *p*-value of each group at phylum level have shown in Supplementary Table S5. Eight genus-level taxons making up more than 1% relative abundance were identified, with *Akkermansia*, *Dubosiella*, *Ileibacterium*, and *Prevotellaceae_NK3B31_group* being the most prevalent. *Akkermansia* abundance slightly increased in the Ferment group, but this difference was not statistically significant (*p* > 0.05). *Ileibacterium* showed an increased trend while *Dubosiella* were reduced. Further, *Bacteroides*, the dominant flora following DSS administration, were significantly lower in the Ferment group. No difference was detected in the other identified genera, including *Lactobacillus* and *Prevotellaceae_UCG-001* after Ferment treatment (Figure 4D). And the *p*-value of each group at genus level have shown in Supplementary Table S6.

Finally, we compared high-dimensional categories using the linear discriminant analysis of effect size (LEfSe). This analysis identified a significant difference in the dominance of bacterial communities between the two groups prior to DSS treatment. *Dubosiella* had the highest LDA score of 4.79 (*p* = 0.0026) in the control group, whereas *Ileibacterium* had the highest LDA score of 4.18 (*p* = 0.0026), and *Prevotellaceae* had the second highest LDA score of 4.22 (*p* = 0.032). These organisms may promote the observed colitis remission mediated by Ferment (Figure 4E). Finally, we determined that *Gluconobacter* and *Lactobacillus* were the dominant flora present in Ferment.

Altogether, Ferment dramatically changed the structure and composition of the murine microbiota.

### 3.4 Therapeutic effect of Ferment on enteritis in mice

**Figure 5:**
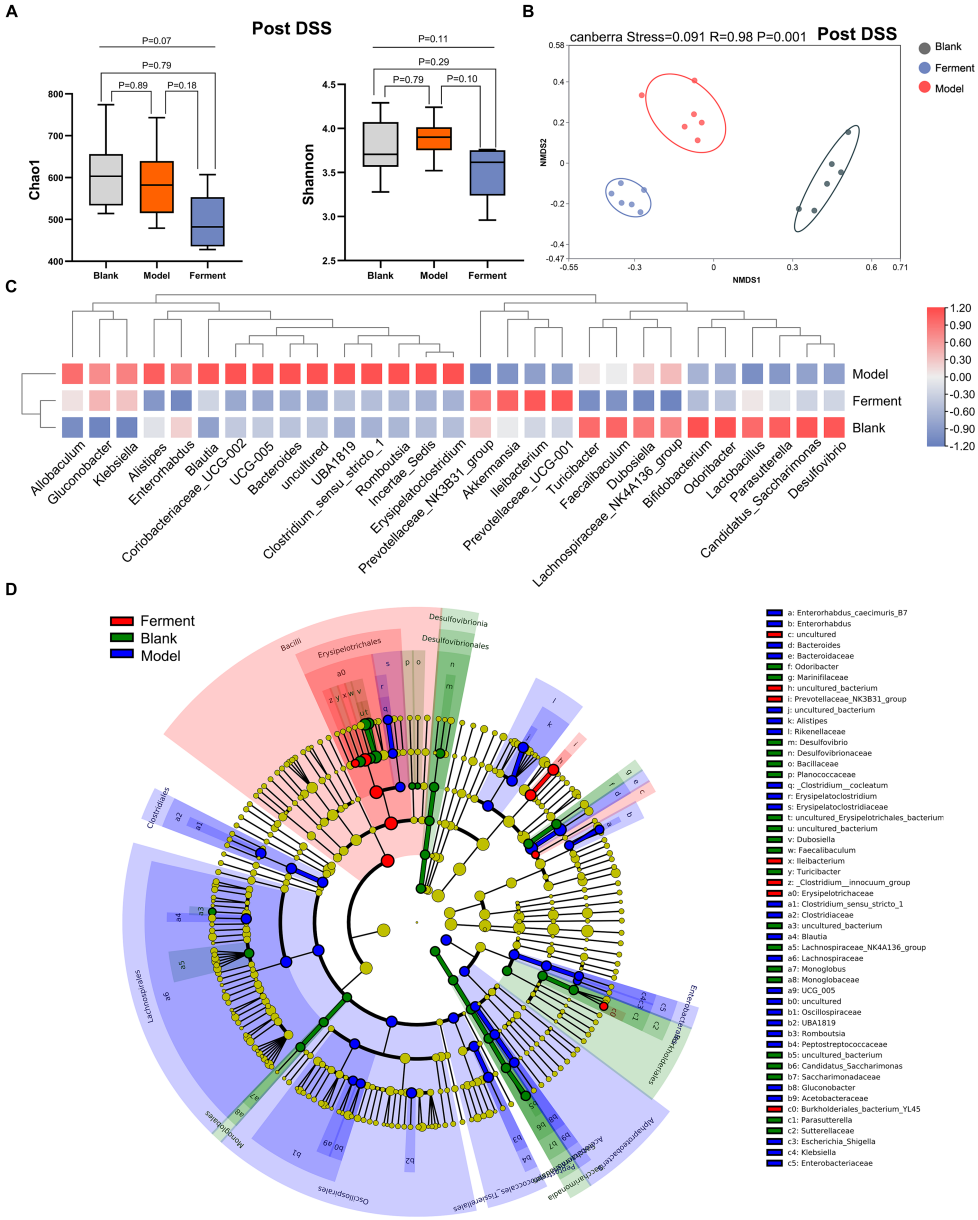
16S rRNA sequencing reveals the therapeutic efficacy of Ferment after Dextran Sulfate Sodium Salt (DSS) treatment. **(A)** α-diversity Chao1 and Shannon indices in Ferment, control, and DSS animals after DSS administration. **(B)** Non-Metric Multidimensional Scaling plot based on Euclidean distance in each sample of control, Ferment and DSS mice after DSS administration. **(C)** Heatmap showing different identified bacteria in control, Ferment and DSS mice following DSS treatment. **(D)** Linear discriminant analysis of effect size cladogram plot of control, Ferment and DSS mice following DSS treatment. ^*^p < 0.05, ^**^p < 0.01, ^***^p < 0.001, and ^****^p < 0.0001.

To identify the richness and diversity of the intestinal microflora, we characterized the α- and β-diversity of fecal samples in the three groups at the post-DSS time point. We observed no difference between the three groups when considering α-diversity (Figure 5A). Instead, NMDS highlighted a large difference between all three groups (*p* < 0.05) (Figure 5B). Next, we employed a heatmap to define the composition of the three groups of flora. *Akkermansia, Prevotellaceae_NK3B31_group, Ileibacterium*, and *Prevotellaceae_UCG-001* were enriched in the Ferment group, and reduced in the DSS group. *Alistipes, Bacteroides, Romboutsia,* and *Erysipelatoclostridium* were enriched in DSS group (Figure 5C). Finally, we employed LEfSe to analyze all three groups. *Ileibacterium* had the highest LDA score of 5.09 (*p* = 0.0005), and *Prevotellaceae_NK3B31_group* had the second highest LDA score of 4.07 (*p* = 0.0033) in the Ferment group. *Bacteroides* had the highest LDA score of 4.95 (*p* = 0.0247), and *Alistipes* had the second highest LDA score of 3.90 (*p* = 0.0046) in the DSS group (Figure 5D; Supplementary Figure S3A). *Proteobacteria* increased in the DSS group and reduced in the Ferment group, whereas *Firmicutes* displayed the opposite trend (Supplementary Figure S3B). Overall, Ferment treatment significantly transformed the diversity and composition of the intestinal microflora.

## 4 Discussion

The incidence of UC is increased in patients with unhealthy living habits or diet, specific genetic factors, abnormal immune functions, and changes to their intestinal flora (Ng et al., 2017). At present, therapeutic management of UC is dominated by available drugs and surgical intervention (Pithadia and Jain, 2011). However, the side effects and high costs of these treatments provide a mandate for the development of alternate therapies. The effect of the gut microbiota on host physiology has been demonstrated through links between gut microbiota dysfunction and host metabolic abnormalities. The therapeutic administration of probiotics, prebiotics, and synbiotics (Akutko and Stawarski, 2021), in addition to fecal microbiota transplantation (FMT) (Zhang et al., 2020), all restore balance to the gut microbiota, and can be considered effective treatments (Khan et al., 2019). Further, studies have demonstrated that after fecal microbiota transplantation (FMT) in UC patients, the synthesis of short-chain fatty acids and secondary bile acids significantly increased. Further, *Eubacterium hallii* and *Roseburia inulivorans* were enriched in patients in remission from UC (Paramsothy et al., 2019). These data suggest that modulating the gut microbiota and associated metabolic pathways is a promising strategy for the treatment of UC.

Animal models of experimental colitis have been developed to investigate the molecular and cellular mechanisms leading to IBD (Chassaing et al., 2014). These models have been employed to develop and evaluate novel anti-inflammatory drugs. In this study, we used the DSS colitis model to characterize the effects of Ferment on UC mice. The main pathological changes in DSS-treated mice were inflammatory lesions of the sigmoid colon, rectal mucosa and submucosa, which was consistent with the pathological changes displayed in human UC disease (Kim et al., 2012). We therefore evaluated the effect of Ferment on both clinical manifestation and pathology in UC mice. Our data show that Ferment was significantly protective against DSS-induced colitis. Ferment-treated mice did not significantly lose weight, had reduced rates of diarrhea, a lower endoscopic index of colitis severity and inflammation, and improved tissue pathology. These data indicate that fermented products can alleviate symptoms and even cure UC.

There are many mechanistic triggers of UC, including altered electrolyte transport in the intestine, compromised intestinal epithelial barriers, oxidative stress, changes to the intestinal flora (Anbazhagan et al., 2018), reduction of goblet cells and associated mucin, and the decreased expression of epithelial NLRP6 (Alipour et al., 2016). In this study, we investigated changes to the gut microbiota. The intestinal microflora, including symbiotic bacteria, pathogens, and probiotics, play an important role in human health. Most of the native flora colonize the intestinal mucosa, attaching to the surface of intestinal epithelial cells to form a biofilm, which ultimately affects the intestinal metabolism of nutrients, intestinal permeability, and immune system function (Veerachamy et al., 2014). Furthermore, probiotics decrease levels of pro-inflammatory cytokines, such as TNF-α and IL-1β, and increase anti-inflammatory IL-10 through the PI3K/Akt and NF-κB signaling pathway, thereby improving symptoms of UC (Shen et al., 2018). To identify changes in the gut microbiota, we performed 16s sequencing, and identified that patterns of change in IBD patients are largely similar, and are characterized by a reduction in gut microbiota diversity. We found a relative increase in the levels of *Proteobacteria* and a relative decrease in the levels of *Firmicutes* in IBD patients, and this strongly correlated with IBD severity, which was in accordance with previous studies (Zhou et al., 2018). Furthermore, *Bacteroides,* which was the key taxon characterizing DSS-treated mice, was also reported as detrimental in previous studies (Wang C. et al., 2019; Li et al., 2021). Notably*, Clostridium_sensu_stricto_1*, which may cause epithelial inflammation (Wang et al., 2018), was also enriched in the DSS-treated group. These observations indicate that the IBD mouse model was successfully implemented in our study.

We found that beneficial organisms were enriched after Ferment treatment. *Ileibacterium* abundance increased after Ferment treatment, indicating that Ferment can improve intestinal flora in UC mice (Wang et al., 2021). Further, the well-known beneficial microorganism *Akkermansia* was enriched after Ferment treatment (Cani et al., 2022). *Prevotellaceae* were also enriched in the Ferment group, and are considered important gut flora (Ley, 2016). Surprisingly, levels of harmful organisms decreased following Ferment treatment. For example, levels of *Erysipelatoclostridium*, a detrimental taxon in DSS colitis mice (Xu et al., 2021), decreased following Ferment treatment. Levels of *Dubosiella,* which can cause radiation enteritis (RE) (Li et al., 2020), were also reduced following treatment. Additionally, *Dubosiella* positively correlated with butyric acid levels, and showed a negative correlation with the mRNA expression of *IL-1β*, *IL-6*, and *TNF-α*, and a positive correlation with *IL-10* expression (Wan et al., 2021). Levels of *Alistipes* were also reduced by Ferment. A previous study identified an altered gut microbiome in Fucosyltransferase 2 (FUT2) loss-of-function mutants and indicated that *Alistipes* was a detrimental constituent of the gut flora in this system. Interestingly, *Alistipes* induced CD8^+^ T cells during IBD. Counterintuitively, butyrate-producing bacteria pose a greater risk to the gut, and as such, *Alistipes* were more abundant in IBD mice (Cheng et al., 2021). Supporting our observations, the species *Alistipes okayasuensis* has already been demonstrated to be pathogenic in the DSS colitis model. Epithelial disruption induced by DSS led to significantly increased levels of inflammatory CD64^+^CD11c^+^ monocytes/macrophages in the lamina propria compartment of the large intestine of mice colonized with a single detrimental flora (Forster et al., 2022), highlighting an avenue for further research. The evidence presented above place Ferment as a cure or preventative therapy for IBD, which functions by increasing beneficial flora, and reducing harmful flora.

Notably, there were two unique favorable floras present in Ferment: *Gluconobacter* and *Lactobacillus. Gluconobacter* is found as a probiotic in fresh unfermented coconut juice (Gopal et al., 2021) and is considered a health-associated probiotic in fermented food (Leech et al., 2020). Studies have reported that *Lactobacillus acidophilus* gavage reduced the levels of pro-inflammatory cytokines, promoted the increase of goblet cells and the secretion of antimicrobial peptides, regulated the ratio of *Firmicutes*/*Bacteroidetes*, and increased the level of acetate (Wang et al., 2020). Furthermore, chitosan was reported to effectively alleviate symptoms in a mouse model of DSS-induced UC. Chitosan improved intestinal mucosal barrier function and promoted the dominant intestinal microflora including *Lactobacillu*s. This activity mitigated intestinal microflora dysbiosis, and regulated the expression of TNF-α, and the tight junction proteins claudin-1, occludin, and ZO-1 (Wang J. et al., 2019). In future studies, we will administer FMT to germ-free mice to identify mechanisms of protection, and characterize how favorable bacteria alter host physiology, and how unfavorable bacteria damage the host.

In conclusion, Ferment can effectively alleviate symptoms in a murine model of DSS-induced UC. Moreover, the intestinal microflora composition in UC mice was distinct from control animals, and Ferment treatment effectively mitigated intestinal microflora dysbiosis. These findings highlight Ferment as a potential agent to ameliorate the severity of UC. Ferment can therefore be developed as an effective food and healthcare product for the prevention of UC and restoration of a balanced intestinal microflora. The stability of probiotics in the treatment of UC, however, still requires improvement. Studies have shown that probiotics can increase gastrointestinal motility, induce diarrhea in IBD patients, alter bowel movements, and increase disease severity (Agathou and Beales, 2013). Severe damage to the intestinal epithelium was reported in a patient undergoing probiotic treatment (Meini et al., 2015), and after 13 days of oral administration, the patient’s condition had worsened, and they began to present with bacteremia, with presence of *L. ramnosus* and *Candida albicans* in the peripheral blood (Jakubczyk et al., 2020). These data suggest that probiotic treatment should be considered on a patient-by-patient basis for each case of colitis. In the future, we will continue to explore how Ferment alleviates UC by altering the microbiota, and will strive to eliminate potential adverse reactions, optimizing Ferment into a probiotic drink for use in large-scale production.

## Data availability statement

The datasets presented in this study can be found in online repositories. The names of the repository/repositories and accession number(s) can be found at: NCBI - PRJNA1082100.

## Ethics statement

The animal study was approved by Nanfang Hospital Animal Ethics Committee. The study was conducted in accordance with the local legislation and institutional requirements.

## Author contributions

YC: Writing – original draft, Writing – review & editing. JS: Writing – review & editing. HW: Writing – review & editing. GD: Writing – review & editing. GW: Writing – review & editing. SW: Writing – review & editing. LY: Writing – original draft, Writing – review & editing. TM: Writing – original draft, Writing – review & editing. SY: Writing – review & editing. QY: Writing – review & editing.

## Footnotes

^1^http://cloud.magigene.com
