# Supplementary material for: Functional evaluation of pure natural edible Ferment: protective function on ulcerative colitis
Source: Front Microbiol. 2024 Jun 17;15:1367630. doi: 10.3389/fmicb.2024.1367630 (PMC11215050; doi:10.3389/fmicb.2024.1367630)
Supplement: Supplementary file 1 [file Data_Sheet_1.docx]

**Functional evaluation of pure natural edible Ferment: protective function on ulcerative colitis**

Yanjun Chen, B.M. ^1†^, Jiaolong Shi, M.D., Ph.D. ^1†^, Hao Wang, M.D., Ph.D. ^2^, Guangxu Deng, M.D., Ph.D. ^3^, Guangxian Wang, Ph.D.^4^, Shijie Wang, Ph.D.^5,6^, Lunan Yang, M.D., Ph.D. ^7#^, Tingyu Mou, M.D., Ph.D. ^1#^

^1^ Department of General Surgery & Guangdong Provincial Key Laboratory of Precision Medicine for Gastrointestinal Cancer, Nanfang Hospital, Southern Medical University, Guangzhou 510515, China.

^2^ First Department of Gastrointestinal Surgery, Hainan General Hospital, Hainan Affiliated Hospital of Hainan Medical University, Haikou 570311, China

^3^ Department of Gastrointestinal and Anorectal, The First People’s Hospital of ZhaoQing,ZhaoQing,China,526000

^4^ Jiangsu Biodep Biotechnology Co., Ltd. Dongsheng West Road, Jiangyin 214400, China

^5^College of Foods Science and Biology, Hebei University of Science and Technology, Shijiazhuang,China,

^6^Junlebao Dairy Group Co., Ltd., Shijiazhuang, Hebei, China,

^7^ Department of Plastic and Aesthetic Surgery, Nanfang Hospital, Southern Medical University, Guangzhou, Guangdong 510515, China

^#^ **Correspondence:**

**Tingyu Mou and Lunan Yang**

Address: Department of General Surgery, Nanfang Hospital, Southern Medical University, 1838, North Guangzhou Avenue, Guangzhou 510515, China.

Email: gary_mou@163.com (**Tingyu Mou**)

Address: Department of Plastic and Aesthetic Surgery, Nanfang Hospital, Southern Medical University, Guangzhou, Guangdong 510515, China

Email: [lunanyang@smu.edu.cn](mailto:lunanyang@smu.edu.cn) (**Lunan Yang**)

† Yanjun Chen and Jiaolong Shi contributed equally and share first authorship.

| **Supplementary Table.1 Physical and chemical index test results** | | | | | |  |
| --- | --- | --- | --- | --- | --- | --- |
|  |  |  |  |  |  |  |
| Indicators | Chinese white pear | Aronia Berry | Red heart pitaya | Green cabbage | Ferment |  |
| Acidity g/kg | 1.08 | 11.61 | 1.62 | 0.89 | 2.79 |  |
| Reducing sugar g/100g | 13.34 | 14.78 | 13.89 | 2.28 | 15.72 |  |
| Soluble solids | 10.09 | 14.2 | 8.85 | 4.85 | 10.08 |  |
| PH | 4.62 | 3.55 | 5.06 | 6.47 | 4.38 |  |
| Polyphenol mg/mL | 0.11 | 0.63 | 0.28 | 0.2 | 0.25 |  |
| Flavone mg/mL | 7.56 | 14.82 | 35.06 | 1.33 | 4.72 |  |
| Tannins mg/L | 134.44 | 464.29 | 301.57 | 221.27 | 296.72 |  |
| Anthocyanins mg/L | 50.62 | 319.15 | 133.79 | 2.29 | 52.18 |  |
| Glucosinolate mg/g | 1.79 | 3.03 | 4.1 | 5.09 | 2.38 |  |

| **Supplementary Table.2 Organic acid test results** | | | | | | |  |
| --- | --- | --- | --- | --- | --- | --- | --- |
|  |  |  |  |  |  |  |  |
| Name | | Chinese white pear (g/L) | Aronia Berry (g/L) | Red heart pitaya (g/L) | Green cabbage (g/L) | Ferment (g/L) |  |
| Oxalic acid | | 1.12 | 0.62 | 0.36 | / | 0.95 |  |
| Tartaric acid | | / | 0.91 | 0.58 | 0.33 | 0.29 |  |
| Pyruvate | | 0.21 | 0.64 | 0.45 | 0.46 | 0.37 |  |
| Shikimic acid | | 0.34 | 1.32 | 0.44 | 0.62 | 0.43 |  |
| L-malic acid | | 0.13 | 0.85 | / | 0.75 | 0.27 |  |
| Lactic acid | | 0.06 | 0.11 | 1.02 | 0.44 | 0.05 |  |
| Acetic acid | | 0.17 | 0.95 | 0.45 | / | 0.21 |  |
| Citric acid | | 0.14 | 1.14 | 0.12 | / | 0.11 |  |
| D-malic acid | | 0.27 | 0.55 | 1.8 | 0.66 | 0.13 |  |
| Succinic acid | | 0.25 | 0.24 | / | / | 0.22 |  |
| Total | | 2.69 | 7.33 | 5.22 | 3.26 | 3.03 |  |
|  |  |  |  |  |  |  |  |
|  |  |  |  |  |  |  |  |

| **Supplementary Table.3 Volatile Substance Test Results** | | | | | | |  |
| --- | --- | --- | --- | --- | --- | --- | --- |
|  |  |  |  |  |  |  |  |
| Name | Chemical Formula | Chinese white pear (mg/L) | Aronia Berry (mg/L) | Red heart pitaya (mg/L) | Green cabbage (mg/L) | Ferment (mg/L) |  |
| Isoamyl alcohol | C_5_H_12_O | / | / | 2.1 | 10.09 | / |  |
| 2/Ethylhexanol | C_8_H_18_O | 2.52 | 3.57 | / | / | / |  |
| Benzyl alcohol | C_7_H_8_O | / | 60.34 | / | / | / |  |
| Phenylethanol | C_8_H_10_O | / | 10.3 | / | 19.07 | 1.26 |  |
| Menthol | C_10_H_20_O | 0.84 | 1.89 | / | / | / |  |
| 1/ Pentadecanol | C_15_H_32_O | / | / | 22.28 | / | / |  |
| 3/ Phenylpropanol | C_9_H_12_O | / | 14.51 | / | / | / |  |
| 1/Octanol | C_8_H_18_O | / | / | / | 1.68 | / |  |
| n-hexanol | C_6_H_14_O | 1.26 | / | 19.34 | / | / |  |
| 5/methyl/2/heptanol | C_8_H_18_O | / | / | / | / | 1.26 |  |
| 1/Octanol | C_8_H_18_O | / | / | / | / | 0.84 |  |
| L/menthol | C_10_H_20_O | / | / | / | / | 0.63 |  |
| Dodecanol | C_12_H_26_O | / | / | / | / | 0.84 |  |
| 1/nonanol | C_9_H_20_O | / | / | 1.68 | / | / |  |
| Undecenol | C_11_H_22_O | / | / | 0.63 | / | / |  |
| 2/tetradecanol | C_14_H_30_O | / | / | 1.47 | / | / |  |
| **Alcohols** |  | 4.62 | 90.61 | 47.5 | 30.84 | 4.83 |  |
| Ethyl acetate | C_4_H_8_O_2_ | / | 8.41 | / | / | / |  |
| Ethyl acrylate | C_5_H_8_O_2_ | 1.26 | / | / | / | / |  |
| Ethyl butyrate | C_6_H_12_O_2_ | 9.88 | 5.68 | / | / | 7.36 |  |
| Ethyl n-hexanoate | C_8_H_16_O_2_ | 4.63 | / | / | / | 2.1 |  |
| Hexyl acetate | C_8_H_16_O_2_ | 1.89 | / | / | / | 0.84 |  |
| 3/ Ethyl methylthiopropionate | C_6_H_12_O_2_S | 23.55 | / | 1.68 | / | 12.4 |  |
| DL-3-ethyl acetate butyrate | C_8_H_14_O_4_ | 1.05 | / | / | / | / |  |
| Ethyl benzoate | C_9_H_10_O_2_ | / | 5.47 | / | / | 1.26 |  |
| 3/ Ethyl Hydroxyhexanoate | C_8_H_16_O_3_ | 4.2 | / | / | / | / |  |
| benzyl acetate | C_9_H_10_O_2_ | / | 6.73 | / | / | 0.84 |  |
| Dihydrokiwifruit lactone | C_11_H_16_O_2_ | / | 3.99 | / | / | / |  |
| Diethyl phthalate | C_12_H_14_O_4_ | 2.31 | 2.73 | 1.26 | / | / |  |
| Diisobutyl phthalate | C_16_H_22_O_4_ | / | / | / | 0.02 | / |  |
| Dibutyl phthalate | C_16_H_22_O_4_ | / | 57.18 | 6.94 | / | 24.39 |  |
| Ethyl palmitate | C_18_H_36_O_2_ | / | 0.84 | / | / | / |  |
| Methyl Salicylate | C_8_H_8_O_3_ | / | / | / | 0.13 | / |  |
| Butyl Butyrate | C_8_H_16_O_2_ | 1.89 | / | 1.26 | / | / |  |
| Dimethyl phthalate | C_10_H_10_O_4_ | 1.47 | / | / | 4.9 | 2.1 |  |
| **Esters** |  | 52.13 | 91.03 | 11.14 | 5.05 | 51.29 |  |
| 3/ Octanone | C_8_H_16_O | 18.92 | 13.45 | 28.38 | / | / |  |
| Acetophenone | C_8_H_8_O | 2.1 | 3.15 | / | / | 1.89 |  |
| damascone | C_13_H_18_O | / | 13.67 | / | 2.21 | / |  |
| β/ionone | C_13_H_20_O | / | 3.36 | / | / | / |  |
| **Ketones** |  | 21.02 | 33.63 | 28.38 | 2.21 | 1.89 |  |
| Hexanal | C_6_H_12_O | 5.47 | / | 1.68 | / | / |  |
| Benzaldehyde | C_7_H_6_O | / | 15.74 | / | / | 8.62 |  |
| Trans/2,4/decadienal | C_10_H_16_O | 0.84 | / | / | / | 7.36 |  |
| **Aldehydes** |  | 6.31 | 15.74 | 1.68 | / | 19.76 |  |
| **Total** |  | 84.08 | 231.01 | 88.7 | 38.1 | 77.77 |  |
|  | | | | | | |  |
|  |  |  |  |  |  |  |  |

| **Supplementary Table.4 Raw material and product polyphenol determination results** | | | | | | |  |
| --- | --- | --- | --- | --- | --- | --- | --- |
|  |  |  |  |  |  |  |  |
| Name | m/Z | Chinese white pear (μg/ml) | Aronia Berry (μg/ml) | Red heart pitaya (μg/ml) | Green cabbage (μg/ml) | Ferment  (μg/ml) |  |
| Cyanidin 3-O-glucoside | 449/287 | 0.11 | 0.13 | 0.1 | / | 0.16 |  |
| Neochlorogenic acid | 335/163 | 1.5 | 0.92 | 1.32 | 1.23 | 1 |  |
| Astilbin | 449/151 | 1.97 | 2.52 | 2.1 | 1.82 | 1.55 |  |
| Resveratrol | 243/159 | 0.9 | 1.7 | 1.33 | 1.21 | 0.88 |  |
| Naringenin | 271/107 | 0.98 | 0.52 | 0.94 | 0.95 | 0.78 |  |
| Quercetin | 303/153 | 0.96 | 0.92 | 0.84 | 1.08 | 0.8 |  |
| Quercetin 3-O-hexoside | 465/303 | 0.62 | 0.43 | / | 0.71 | 0.52 |  |
| Catechin | 291/139 | 0.11 | 0.24 | 0.23 | 0.12 | 0.18 |  |
| p-coumaric acid | 165/119 | 0.04 | 0.25 | 0.22 | 0.05 | 0.06 |  |
| Kaempferol-7-O-glucoside | 447/179 | 0.38 | 0.79 | 0.54 | 0.92 | 0.36 |  |
| Total |  | 7.57 | 8.42 | 7.62 | 8.09 | 6.29 |  |
|  | | | | | | |  |
|  |  |  |  |  |  |  |  |

**Supplementary Table.5 P-value at the phylum level for the Ferment and Blank groups**

| Phylum | Sub-Group | P-value | Average-value | 95% CL | |
| --- | --- | --- | --- | --- | --- |
|  |  |  |  | Low | High |
| Firmicutes | Ferment | 0.218 | 0.355 | -0.262281831 | 0.690004087 |
|  | Blank |  | 0.451 |  |  |
| Bacteroidota | Ferment | 0.263 | 0.329 | -0.570357539 | 0.188157752 |
|  | Blank |  | 0.264 |  |  |
| Verrucomicrobiota | Ferment | 0.347 | 0.285 | -0.617936429 | 1.605200815 |
|  | Blank |  | 0.235 |  |  |
| Actinobacteriota | Ferment | 0.130 | 0.013 | -0.170777051 | 0.002516861 |
|  | Blank |  | 0.019 |  |  |
| Campilobacterota | Ferment | 0.831 | 0.007 | -0.249294968 | 0.020583451 |
|  | Blank |  | 0.009 |  |  |
| Patescibacteria | Ferment | 0.089 | 0.001 | -0.020240132 | 0.00189643 |
|  | Blank |  | 0.010 |  |  |
| Desulfobacterota | Ferment | 0.700 | 0.005 | -0.007117664 | 0.004960203 |
|  | Blank |  | 0.006 |  |  |
| Proteobacteria | Ferment | 0.003 | 0.005 | 0.00128178 | 0.004772976 |
|  | Blank |  | 0.002 |  |  |

**Supplementary Table.6 P-value at the genus level for the Ferment and Blank groups**

| Genus | Sub-Group | P-value | Average-value | 95% CL | |
| --- | --- | --- | --- | --- | --- |
|  |  |  |  | Low | High |
| Akkermansia | Ferment | 0.347 | 0.285 | 0.049363 | 0.050112 |
|  | Blank |  | 0.235 |  |  |
| Dubosiella | Ferment | 0.006 | 0.039 | -0.121808 | 0.030287 |
|  | Blank |  | 0.162 |  |  |
| Ileibacterium | Ferment | 0.008 | 0.152 | 0.126011 | 0.030945 |
|  | Blank |  | 0.026 |  |  |
| Prevotellaceae_NK3B31_group | Ferment | 0.033 | 0.059 | 0.031577 | 0.012956 |
|  | Blank |  | 0.028 |  |  |
| Lactobacillus | Ferment | 0.049 | 0.016 | -0.021725 | 0.009334 |
|  | Blank |  | 0.038 |  |  |
| Faecalibaculum | Ferment | 0.006 | 0.004 | -0.033469 | 0.008224 |
|  | Blank |  | 0.038 |  |  |
| Prevotellaceae_UCG-001 | Ferment | 0.106 | 0.007 | -0.009802 | 0.005288 |
|  | Blank |  | 0.017 |  |  |
| Bacteroides | Ferment | 0.049 | 0.005 | -0.011942 | 0.00488 |
|  | Blank |  | 0.017 |  |  |


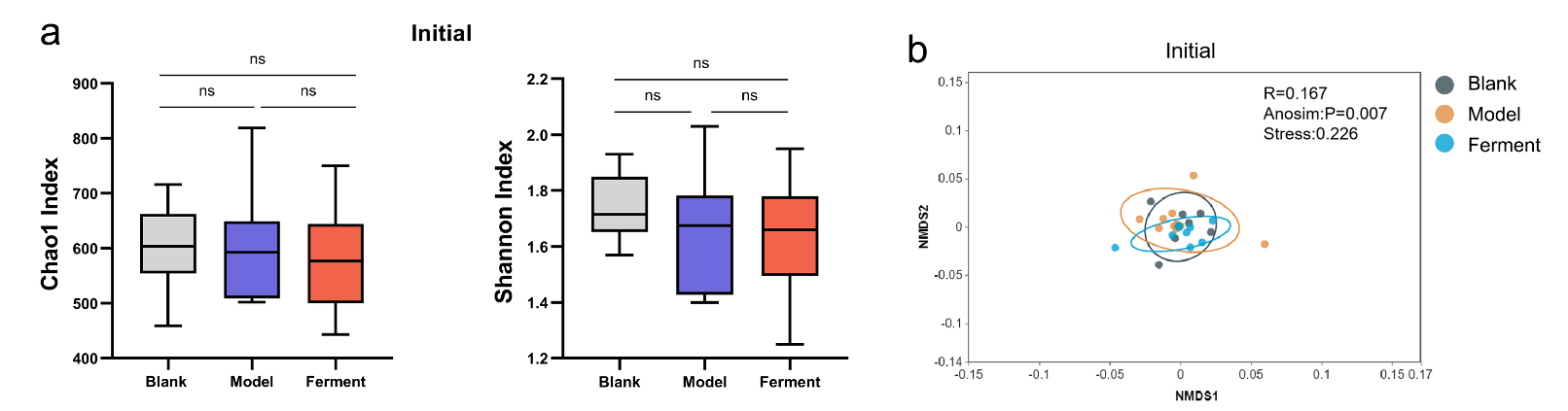
**Supplementary Figure 1**

**Supplementary Figure 1.** (a) α-diversity Chao1 and Shannon indices in control and DSS-treated and Ferment-treated animals at initial time point. (b) Non-Metric Multidimensional Scaling plot based on Canberra dissimilarity in each sample of control and DSS mice and Ferment mice pre at initial time point.

**Supplementary Figure 2**

**
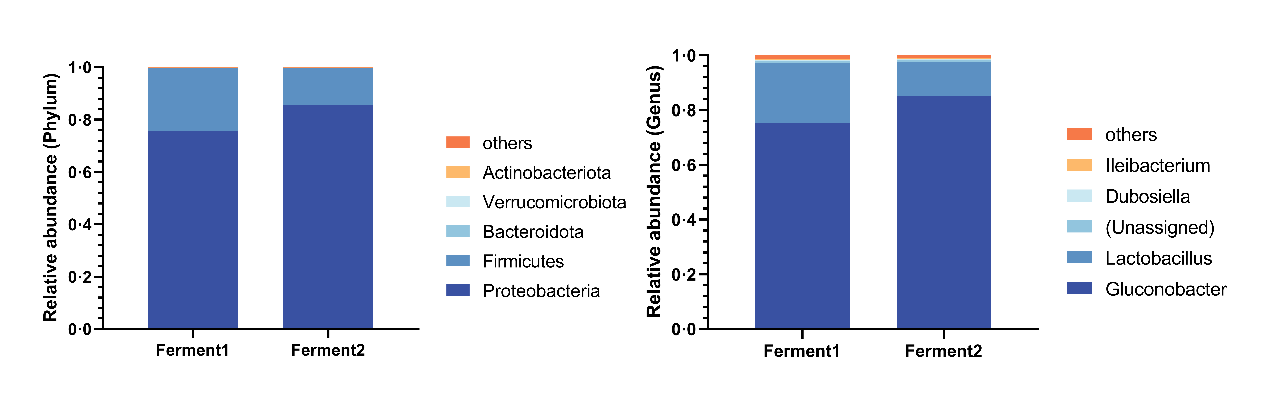
**

**Supplementary Figure 2.** Gut microbiota composition in Ferment drink at the (a) phylum and (b) genus level.

**Supplementary Figure 3**

**
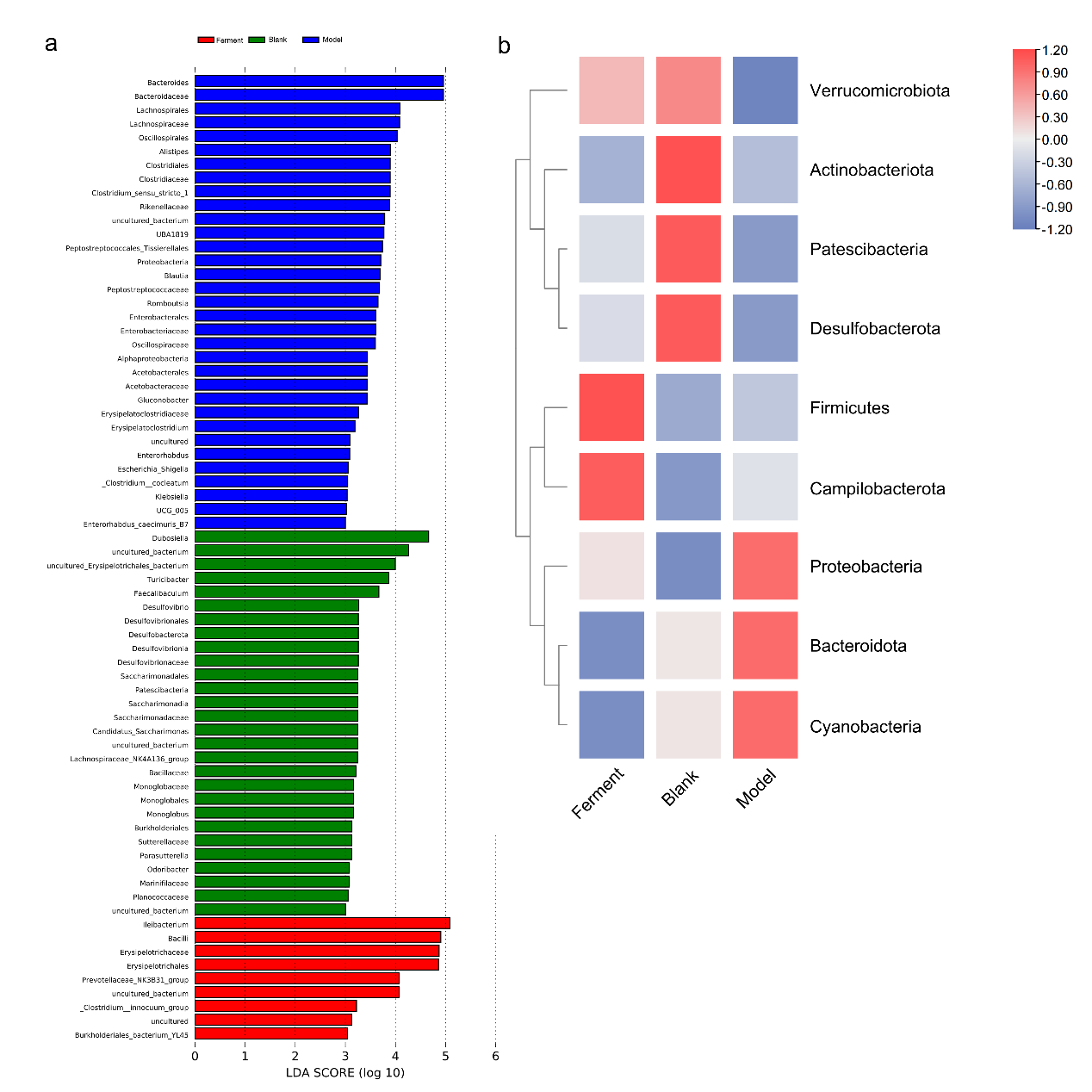
**

**Supplementary Figure 3.** Linear discriminant analysis of effect size cladogram plot of control, Ferment and DSS mice following DSS treatment and compare with Blank group. Heatmap showing different identified bacteria in control, Ferment and DSS mice and Blank group following DSS treatment
